# Uncertainty assessment of PM_2.5_ contamination mapping using spatiotemporal sequential indicator simulations and multi-temporal monitoring data

**DOI:** 10.1038/srep24335

**Published:** 2016-04-12

**Authors:** Yong Yang, George Christakos, Wei Huang, Chengda Lin, Peihong Fu, Yang Mei

**Affiliations:** 1 Department of Resoure and Environmental Information, College of Resources and Environment, Huazhong Agricultural University, Wuhan 430070, China; 2 Key Laboratory of Arable Land Conservation (Middle and Lower Reaches of Yangtse River), Ministry of Agriculture, Wuhan 430070, China; 3 Institue of Island and Coastal Ecosystems, Ocean College, Zhejiang University, Hangzhou, Zhejiang 310027, China; 4 Department of Geography, San Diego State University, San Diego, Califiornia 92182, USA

## Abstract

Because of the rapid economic growth in China, many regions are subjected to severe particulate matter pollution. Thus, improving the methods of determining the spatiotemporal distribution and uncertainty of air pollution can provide considerable benefits when developing risk assessments and environmental policies. The uncertainty assessment methods currently in use include the sequential indicator simulation (SIS) and indicator kriging techniques. However, these methods cannot be employed to assess multi-temporal data. In this work, a spatiotemporal sequential indicator simulation (STSIS) based on a non-separable spatiotemporal semivariogram model was used to assimilate multi-temporal data in the mapping and uncertainty assessment of PM_2.5_ distributions in a contaminated atmosphere. PM_2.5_ concentrations recorded throughout 2014 in Shandong Province, China were used as the experimental dataset. Based on the number of STSIS procedures, we assessed various types of mapping uncertainties, including single-location uncertainties over one day and multiple days and multi-location uncertainties over one day and multiple days. A comparison of the STSIS technique with the SIS technique indicate that a better performance was obtained with the STSIS method.

Numerous studies have indicated that particulate matter (PM) in the atmosphere is related to various adverse impacts on human health^1^,^2^. China has experienced rapid economic growth and industrialization as well as a surge in car usage and urbanization, and these changes have generated severe amounts of particulate matter (PM) pollution^3^ and caused serious health impacts on China’s populace. For example, the statistical data from the National Health and Family Planning Commission of China showed that the current lung cancer incidence rate in China is growing by approximately 26.9% a year^4^. To evaluate the PM pollution conditions in China, the Chinese government has investigated the underlying characteristics of PM pollution. On February 29^th^, 2012, the third revision of the “Ambient Air Quality Standard” (AAQS) (GB 3095-2012) was released^5^, and starting in January 2013, 113 of the major cities in China began releasing the recorded concentrations of seven pollutants, including sulfur dioxide (SO_2_), nitrogen dioxide (NO_2_), particulate matter with aerodynamic diameters equal to or less than 10 μm (PM_10_), particulate matter with aerodynamic diameters equal to or less than 2.5 μm (PM_2.5_), carbon monoxide (CO), 1 h peak ozone (O_3_), and 8 h peak O_3_^6^. Based on these monitoring data, a number of studies have been performed to determine the spatiotemporal variability of pollutants in the air^3^,^7^,^8^. In addition, a number of studies have used spatio-temporal geostatistical methods, including Bayesian maximum entropy (BME)^9^,^10^,^11^ and kriging interpolations^12^, to determine the spatiotemporal distribution of pollutants. However, a smoothing effect commonly occurs in maps generated by these techniques, and it can cause underestimations or overestimations of pollutants^13^ and misclassifications of polluted areas. However, the kriging estimate at each unsampled location includes a kriging variance that measures the estimation uncertainty. A contaminated area cannot be reliably classified without considering this uncertainty^14^; thus, estimation uncertainty is an important factor when assessing the level of risk resulting from a pollutant^15^.

Generally, risk assessments are based on the quantification of specific uncertainties involved in classifying contaminated sites, and the results are expressed in terms of exceedance probabilities. In certain cases, quantitative uncertainty assessments can be performed using two main groups of techniques: the first group includes non-linear geostatistics techniques, such as disjunctive kriging (DK) and indicator kriging (IK)^16^,^17^,^18^; and the second group includes stochastic simulation algorithms, such as sequential indicator simulations (SISs) and sequential Gaussian simulations (SGSs), which generate a set of equiprobable representations (realizations) of the spatial distribution of target attribute values and uses the differences among the simulated maps as a measure of uncertainty^19^,^20^. In general, SIS is more commonly used (or perhaps is more “fashionable”) than IK for uncertainty modeling^21^. Moreover, SIS can overcome the limitations inherent in IK, such as the smoothing effect^22^ and an inability to consider variation in estimations at unsampled locations or simultaneously reproduce multi-points of uncertainty^21^.

However, long-term uncertainty information related to PM in the atmosphere for a region may be more meaningful because a number of studies have linked long-term exposure to PM with certain diseases^23^,^24^. Nevertheless, the uncertainty assessment methods listed above are generally used for processing data in a single period because they are incapable of integrating multi-temporal data. Therefore, we cannot determine the spatial distribution of exceedance probabilities over a long period of time. Furthermore, it is important to determine whether multi-temporal data for an environmental variable can improve the accuracy of uncertainty models when the environmental variable is monitored continuously over many sites.

Based on these considerations, the aim of the present work was to use the spatiotemporal sequential indicator simulation (STSIS) technique^25^ to assimilate multi-period data and generate many realizations. The many realizations generated by STSIS were subsequently used to estimate the various uncertainties associated with the delineation of PM_2.5_, and the results were compared with those obtained using the SIS. For illustration purposes, we used a data set of PM_2.5_ concentrations in the air recorded in 2014.

## Materials and Methods

### Study area and data sources

**Figure 1:**
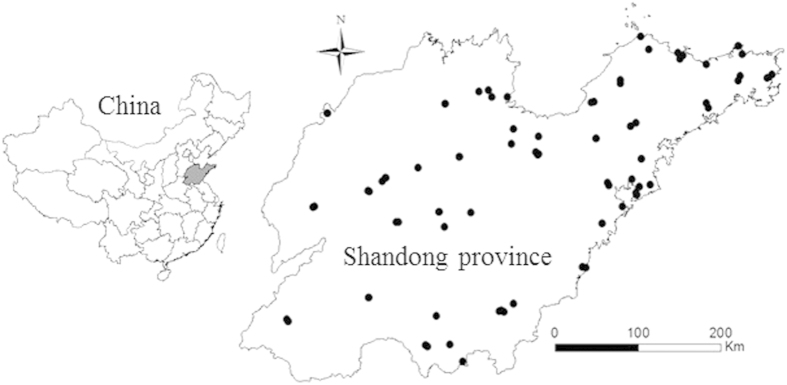
Location of the study area and the spatial distribution of the monitoring sites. (Created by ArcMap, version 10.2, http://www.esri.com/).

The study area is located in Shandong Province, China, and it covers a national territorial area of 157.9 thousand Km^2^. The data presented in this study were obtained from 96 national air quality monitoring sites during the period from January 1, 2014 to December 31, 2014 (data were obtained from the following website: http://113.108.142.147:20035/emcpublish). The spatial distribution of monitorting was shonw in Fig. 1. The ambient concentration of PM_2.5_ was measured according to the China Environmental Protection Standard HJ655-2013^26^. At each site, the daily PM_2.5_ concentration was calculated by averaging the hourly data.

### Spatiotemporal sequential indicator simulation (STSIS) algorithm

To distinguish between space (S) and time (T), let *Z*(x) = {*Z*(*s*, *t*)|*s *∈ *S*, *t *∈ *T*} represent a variable defined on a geographical domain *S *∈ *R*^2^ and a time interval *T*∈R. The STSIS algorithm used in this study involves the following steps. The first step is to code each PM_2.5_ concentration observation value *z*(*s*, *t*) into vector K indicator values using the indicator transformation function *I*(*s*, *t*; *z*_*c*_):


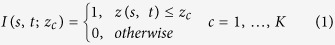


where *z*_*c*_ is a desired cutoff value of PM_2.5_ concentrations. In this study, the cutoff values were set to 34 μg · *m*^−3^ (20% percentile), 53 μg · *m*^−3^ (40% percentile), 74 μg · *m*^−3^ (60% percentile), and 106 μg · *m*^−3^ (80% percentile). For each of the four PM_2.5_ concentration cutoff values (*z*_*c*_), the experimental spatio-temporal (ST) semivariogram of the indicator code was calculated using the following equation:


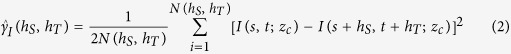


where *h*_*S*_ and *h*_*T*_ are the spatial and temporal lags, respectively, and *N*(*h*_*s*_, *h*_*T*_) is the number of pairs in the ST lag for the indicator codes of PM_2.5_ concentrations.

There are two main approaches to fitting a theoretical model to the spatiotemporal experimental variogram. The first approach relies on separable variogram modeling, which assumes separate spatial and temporal variation structures and represents the total ST variogram as the sum of these structures. This approach facilitates structural analyses; however, it presents a number of important drawbacks caused by assumption of a strict separation of spatial and temporal structures. For example, this approach implies that the spatial behavior must be the same for all time points and the temporal behavior must be the same at all spatial locations. However, such consistency is not observed in practice, where different spatial patterns emerge at different times and time series at different locations show different behaviors^27^,^28^. The second approach relies on a non-separable model that can overcome some of the above drawbacks. There are various non-separable covariance and variogram models^29^,^30^,^31^,^32^. In this study, the experimental ST semivariogram was modeled using a non-separable spatiotemporal semivariogram model.


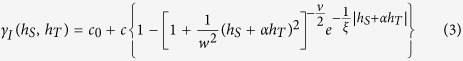


The parameters *c*_0_, *c*, *v*, *w*, *ξ* and *α* should be calculated from the data. The parameters of the model (3) were calculated simultaneously using a genetic algorithm to simultaneously estimate the parameters^33^ using a fitness minimization function, such as the root mean square error (RMSE):


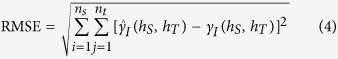


where *n*_*s*_ and *n*_*t*_ denote the number of spatial and temporal data pairs, respectively.

A random path visiting each ST node of a grid defined over the study area was established. Base on the procedures of SIS^34^,^35^, at each unsampled location, the following procedures were employed.

- The probability that the PM_2.5_ concentration will not exceed 34, 53, 74 and 106 μg · *m*^−3^ was estimated at each point (*s*_*i*_, *t*_*i*_) of the random path as a linear combination of the neighboring indicator values using ST ordinary kriging. These probabilities were formally expressed by the corresponding conditional cumulative distribution function (CCDF), whereas the spatiotemporal distances between (*s*_*i*_, *t*_*i*_) and the neighboring indicator data points were determined by *h*_*s*_ + *αh*_*T*_.
- The order relation deviations of the obtained probabilities were corrected, and a continuous model of the prior CCDF of the PM_2.5_ concentration at location (*s*_*i*_, *t*_*i*_) was built by interpolating or extrapolating the CCDF values.
- A simulated PM_2.5_ concentration value was randomly drawn from the prior CCDF at each spatiotemporal point (*s*_*i*_, *t*_*i*_).
- The indicator code of the simulated value at location (*s*_*i*_, *t*_*i*_) was added to the prior CCDF modelling at the next point (*s*_*i*+1_, *t*_*i*+1_).
- Following the random path, the procedure (i)–(iv) above was repeated until all of the nodes were visited and each node was assigned a simulated value, thus obtaining a STSIS realization.

By selecting various random paths, a number of STSIS realizations were generated. Each realization used a different path to visit all of the nodes of the grid covering the study area, thus representing a possible spatiotemporal distribution of PM_2.5_ concentrations. In this way, the mapping uncertainty was determined using a number of STSIS realizations. In the present study, 1000 realizations were generated using STSIS.

## Uncertainty Assessment

### Single location uncertainty for one day

The uncertainty of PM_2.5_ estimation at a single spatiotemporal location **p**′ = (*s*′, *t*′), which indicates that the probability of a PM_2.5_ concentration z(**p**′) is higher than the threshold level of contamination (*z*_*c*_; e.g., 75 μg · *m*^−3^), can be represented by the following exceedance probability:


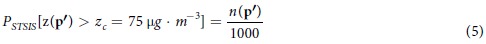


where the threshold value of 75 μg · *m*^−3^ represents the lower limit of light pollution for PM_2.5_ concentrations according to the China National Ambient Air Quality Standards^5^ and *n*(**p**′) is the number of PM_2.5_ realizations generated by STSIS in which the concentration values were greater than the threshold *z*_*c*_ (out of a total of 1000 realizations). The exceedance probability of Eq. (5) expresses the likelihood that a designated value (*z*_*c*_) will be exceeded. In addition, the variance 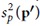 of *P*_*STSIS*_[z(**p**′) > *z*_*c*_] is obtained as follows:


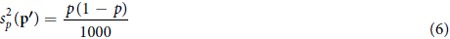


where *p* is the value of 
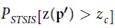
.

### Single-location uncertainty for multi-days

A single-location uncertainty refers to the joint PM_2.5_ estimation uncertainty at a spatial location over multiple days, and it indicates that the probability of PM_2.5_ concentrations z(**p**′) over multi-days may be higher than the contamination threshold *z*_*c*_:


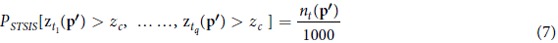


where *n*_*t*_(**p**′) is the number of realizations in which the PM_2.5_ concentrations generated by STSIS over the multi-day period were greater than the threshold in each one of the 1000 realizations. A location with multi-day uncertainties can reveal the pollution risk over the long term. The variance of Eq. (7) can also be obtained from Eq. (6).

### Multi-location uncertainty for one day

A one-day multi-location uncertainty represents the joint uncertainty at several specified locations over a single day, and it can be used to measure the reliability of contamination assessments based on the probability map of *P*_*STSIS*_[z(**p**′) > *z*_*c*_] for a given critical probability *p*_*c*_. For example, for a given *p*_*c*_ and PM_2.5_ concentrations *z*_*c*_, the number of points **p**′ where the following condition applies should be determined:


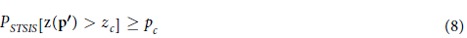


Accordingly, the probability that the PM_2.5_ concentrations at *n* locations in an area will all be greater than the threshold *z*_*c*_ can be calculated based on the following equation:


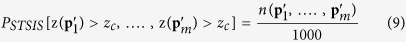


where 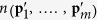 is the number of realizations in which all of the simulated PM_2.5_ values at the *m* locations are greater than *z*_*c*_ (in this case, out of a total of 1000 realizations). The variance can also be calculated as follows:


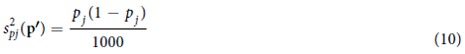


where *p*_*j*_ is the value of the probability in Eq. (9).

### Multi-location uncertainty over multi-days

Multi-day multi-location uncertainties represent the joint uncertainty at a set of specified locations over the multi-day period of interest, and they can be used to assess the reliability of contamination assessments based on the following probability map for a given *p*_*c*_:


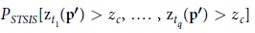


Based on Eq. (9), the multi-day multi-location uncertainty can be calculated as follows:


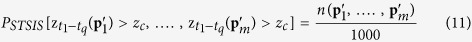


where 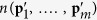 is the number of realizations in which all of the simulated PM_2.5_ concentration values at *m* locations in an area exceed *z*_*c*_ over a multi-day period (out of a total of 1000 realizations). The variance can also be calculated as in Eq. (10).

### Goodness of uncertainty assessment

For comparative analysis purposes, the SIS technique^21^ was used to assess single-location PM_2.5_ uncertainties using data recorded for the same day only. Then, we compared the results obtained by SIS with those obtained by STSIS.

Based on the CCDF *F* (**u**; z|(*n*)) at any test location **u** (where the notation |(*n*) expresses conditioning to the local information, such as *n* neighboring data), the series of symmetric *p* probability intervals (PI) considered were bounded by the corresponding *p*-percentile. For example, the 0.5 PI is expressed as *F*^−1^(**u**; 0.5|(*n*), + ∞] or as *F*^−1^(**u**; 0.5|(*n*), *z*_*max*_] in practice. Adequate local uncertainty modeling requires that 50% of the true values over the study area locally exceed the CCDF median. Given a set of sampling points and independently generated CCDFs by the STSIS and SIS techniques at the corresponding *N* sampling locations **u**_*j*_, 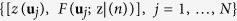, where |(*n*) denotes the conditioning to the local information (e.g., *n* neighboring data), the fraction of true values falling into the symmetric *p* PI was calculated as follows:


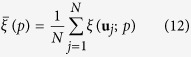


for all *p *∈ [0, 1], with


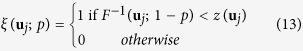


At this point, the root mean squared error (RMSE) for the *T* technique (in this case, *T *= SIS and STSIS) was defined as follows:


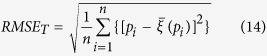


where *T* = SIS and STSIS and 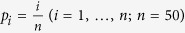. Smaller RMSE values suggest more accurate assessments of PM_2.5_ contamination uncertainty. The true value should fall into the PI according to the expected probability, and this interval should be as narrow as possible to reduce the value’s uncertainty. Therefore, a better probabilistic model would generate a smaller spread (less uncertain). In this study, the average width of the PIs for a series of probabilities *p*, 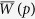, was calculated as follows:


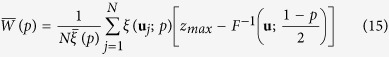


Smaller 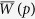 values indicate that the PIs are narrower and the method has greater accuracy.

## Results and Discussion

### Preliminary data description

**Figure 2:**
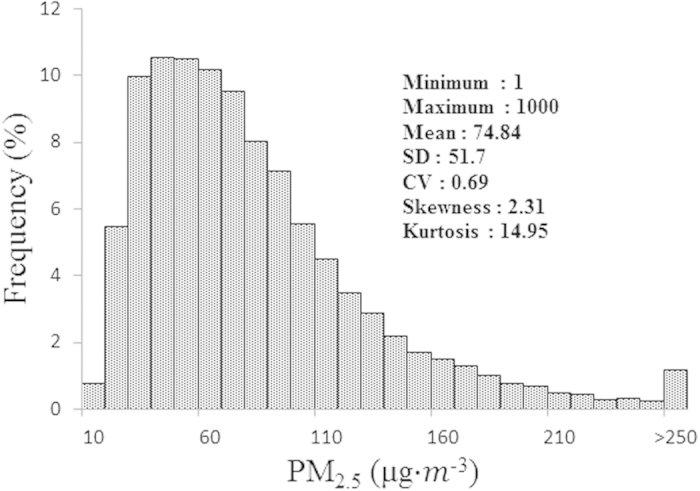
Statistical characteristics of the PM_2.5_ concentrations for all of the collected data from Shandong Province in 2014. (SD, standard deviation; CV, coefficient of variation).

**Figure 3:**
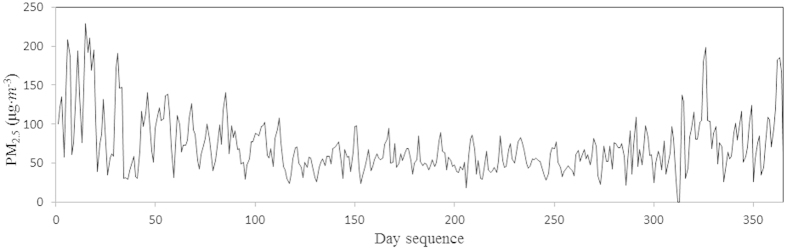
Daily variation of the PM_2.5_ means for all of the monitoring sites from 2014.1.1 to 2014.12.31.

A summary of the descriptive statistics of the PM_2.5_ concentrations recorded in 2014 is presented in Fig. 2. The temporal trend of the average values for all of the monitoring sites is presented in Fig. 3. Figure 2 shows that the PM_2.5_ concentrations for all of the collected data ranged from 1 to 1000, and the mean concentration was 74.84. The coefficient of variation (CV) was 0.69, which indicates that the PM_2.5_ for all of the monitoring data presented a medium variability (i.e., 0.1 < CV < 1). The skewness and kurtosis values were 2.31 and 14.95, respectively, indicating that the null hypothesis of normality was rejected for the monitoring data.

Figure 3 shows that a characteristic seasonal variation in the PM_2.5_ occurs in the study area, with elevated concentrations occurring in spring and winter. These variations are related to seasonal fluctuations in the emissions as well as to meteorological effects^3^,^32^.

### Spatiotemporal indicator semivariograms

**Figure 4:**
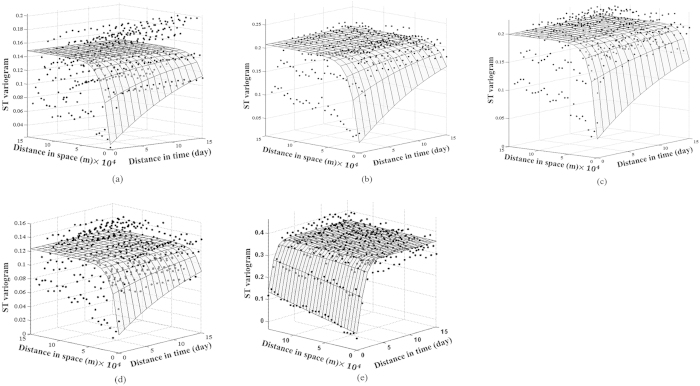
ST indicator semivariograms for (**a**) *z*_*c*1_, (**b**) *z*_*c*2_, (**c**) *z*_*c*3_, and (**d**) *z*_*c*4_, and (**e**) ST semivariograms for Lg(PM_2.5_). Dots represent experimental semivariogram data. Curved surfaces depict the fitted theoretical models.

Eq. (1) was used to obtain the indicator values for all of the original values, and then Eq. (2) was used to calculate the experimental spatiotemporal indicator semivariograms and fit the models of Eq. (3) to the four cutoff values. These models were subsequently used with the STSIS technique to build the prior CCDF. In Fig. 4, the indicator semivariograms of the non-separable models of Eq. (3) are fit to the experimental semivariograms. The values of the model parameters are listed in Table 1.

### Mapping PM2.5 concentrations: STSIS vs. STOK

**Figure 5:**
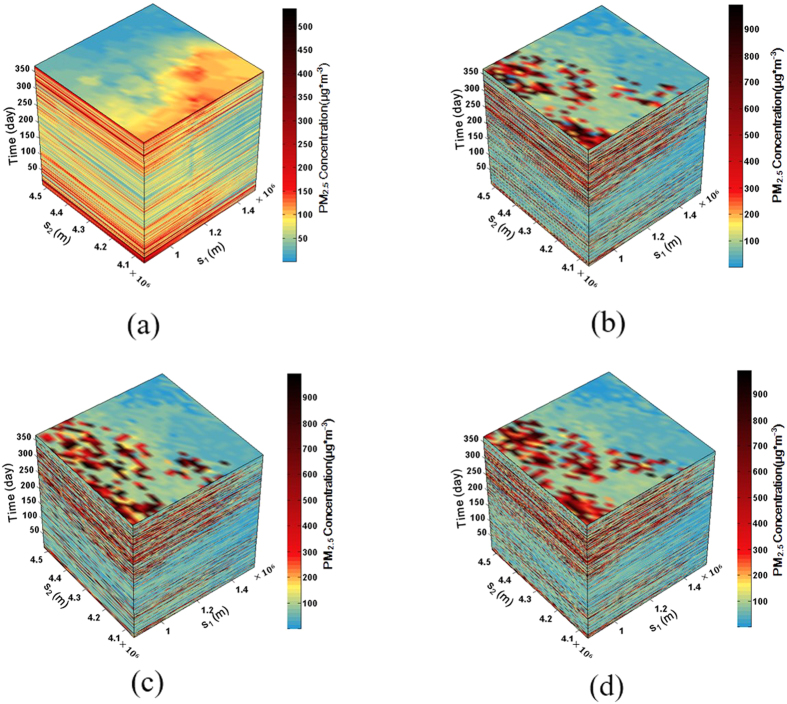
3-D plots of the spatiotemporal distribution of PM_2.5_ obtained by the (**a**) STOK, and (**b–d**) three randomly selected STSIS realizations (out of 1000 realizations).

To compare the results generated by STSIS and a general ST prediction method, spatiotemporal ordinary kriging (STOK)^36^ was employed to predict the ST distribution of PM_2.5_. As shown in Fig. 2, the null hypothesis of normality was rejected for the original monitoring data. Thus, before performing the STOK, the data were logarithmically transformed. After the logarithmical transformation, the K-S test value, the skewness value and the kurtosis value were 4.725, −0.252, and 0.145, respectively. Thus, the PM_2.5_ concentrations after the logarithmical transformation followed a normal distribution. The experimental ST variograms were calculated, and the theoretical model was fit. The results are shown in the last row of Table 1 and Fig. 4(e). A STOK prediction was then performed on the LgPM_2.5_ concentration data based on the ST theoretical variogram model. Finally, the predicted LgPM_2.5_ values were translated into the original values by antilogarithms (Fig. 5(a)). Three randomly selected STSIS realizations out of 1000 realizations are shown in Fig. 5(b–d).

A comparison of the summary statistics is shown in Table 2. The maximum value, the standard deviation (SD) and coefficient of variation (CV) of the STOK were obviously smaller than those of the STSIS and original data, indicating an obvious smoothing effect of the STOK. However, the maximum value, SD and CV of the STSIS realizations were close to those of original data, indicating a similar variability in the STSIS results with that of the original data. As shown in Fig. 5. The STSIS polygons were more fragmented relative to those of the STOK because of the smoothing effect of the STOK. Thus, the STOK results only present a simplistic spatial pattern and do not capture important information that is revealed in the more detailed STSIS maps, such as hot PM_2.5_ spots. Moreover, the STSIS realizations covered all possible spatial patterns, indicating that mapping uncertainties can be fully assessed by using a sufficient number of STSIS realizations.

### Contaminated sited classifications based on single-location uncertainties

**Figure 6:**
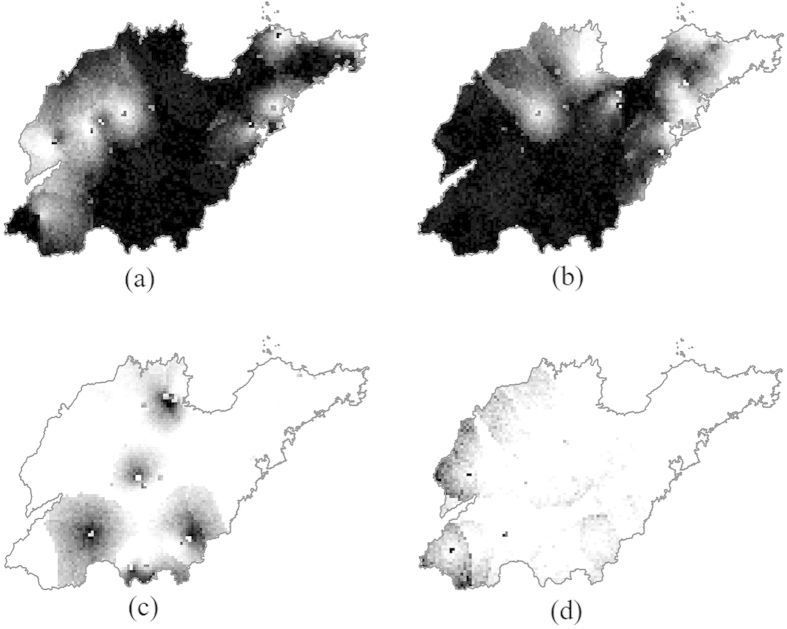
STSIS-generated maps of the PM_2.5_ exceedance probabilities (probabilities of PM_2.5_ concentrations exceeding 75 μg · *m*^−3^) on the (**a**) 1^st^ day, (**b**) 100^th^ day, (**c**) 200^th^ day, and (**d**) 300^th^ day. (Created by ArcMap, version 10.2, http://www.esri.com/).

In this study, 1000 STSIS simulated realizations were used to determine the single-location uncertainties, which are measured on four time scales: one day, one month, one season, and one year. In addition, the uncertainties are expressed by the probabilities of the PM_2.5_ concentrations being higher than a certain threshold value. Figure 6 shows that the probability that the PM_2.5_ concentration will exceed 75 μg · *m*^−3^ for most of the study locations on the 1^st^ day and 100^th^ day is close to 1, whereas the probability that the PM_2.5_ concentration will exceed 75 μg · *m*^−3^ for most of the study locations on the 200^th^ day and 300^th^ day is close to 0. Moreover, the highest probabilities on the 200^th^ day and 300^th^ day are 0.98 and 0.99, respectively, indicating that none of the study sites will present a PM_2.5_ concentration that will definitely exceed 75 μg · *m*^−3^ for these two days.

**Figure 7:**
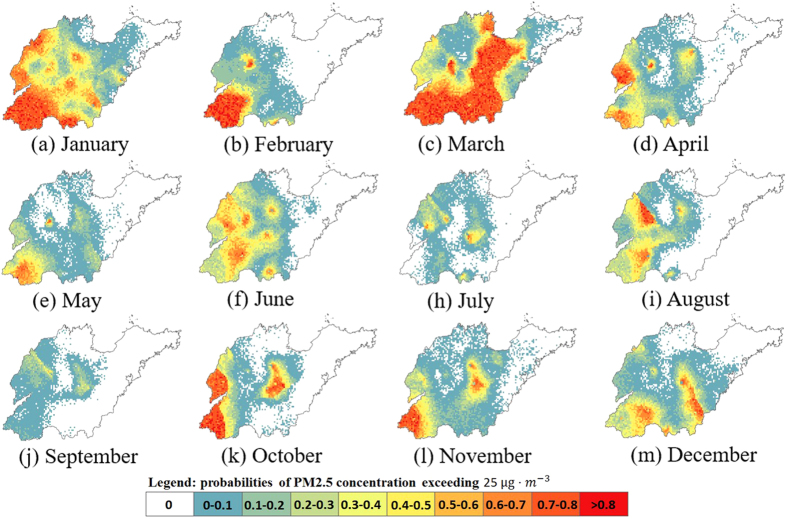
STSIS-generated maps of the PM_2.5_ exceedance probabilities (probabilities of PM_2.5_ concentrations exceeding 25 μg · *m*^*−*3^) in each month of 2014. (Created by ArcMap, version 10.2, http://www.esri.com/).

**Figure 8:**
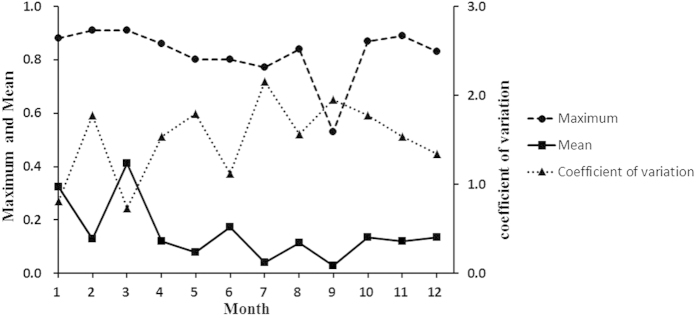
Maximum, mean and coefficient of variation of the PM_2.5_ exceedance probabilities (probabilities of the PM_2.5_ concentration exceeding 25 μg · *m*^*−*3^) for each month of 2014.

Figure 7 shows the spatial distribution of the probabilities in which the PM_2.5_ concentrations will exceed 25 μg · *m*^−3^ (guideline provided by the WHO)^37^ for each month of 2014. These maps show the points with high or low 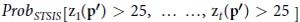 values according to Eq. (7). As shown in Figs 7 and 8, the highest probabilities from January to September are 0.88, 0.91, 0.91, 0.86, 0.8, 0.8, 0.77, 0.84, 0.53, 0.87, 0.89, and 0.83, indicating that there are a number of areas in which the PM_2.5_ concentrations might always exceed 25 μg · *m*^−3^ during each month. The smallest probability is 0, and 12.7%, 46.6%, 8.7%, 37.6%, 41.5%, 33.6%, 54.9%, 47.5%, 58.5%, 50.3%, 28.1%, and 28.8% of the study locations presented a probability of 0 from January to December, indicating that the PM_2.5_ concentrations in these areas are not always >25 μg · *m*^−3^ during the corresponding month. As shown in Fig. 8, the low mean values with high coefficients of variation (CVs) are found in May, July and September, and these values indicate lower PM_2.5_ pollution risks and high variation. Furthermore, the highest mean value and lowest CV are found in March, indicating that the highest PM_2.5_ pollution risk occurs for almost the entire study area in this month. In terms of spatial distribution, a high PM_2.5_ pollution risk (Fig. 7) is observed in the southwestern region of the study area during January, February, March, October, and November; and an absence of PM_2.5_ pollution risk is observed in the eastern region of the study area at the month scale for the entire year.

### Contaminated site classification with multi-location uncertainty

The CCDF generated by the STSIS can be used to measure the local uncertainty at a single location; however, a series of single-point CCDFs cannot be used to measure multi-point spatial uncertainties^21^. Therefore, an adequate reliability assessment of PM_2.5_ contamination distributions requires a multi-location uncertainty assessment for 1 day (multi-location/single-day uncertainty) and multiple days (multi-location/multi-day uncertainty) at a set of locations in the contaminated area based on the corresponding single-location uncertainty for 1 and multi-days (single-location/single-day uncertainty and single-location/multi-day uncertainty, respectively).

**Figure 9:**
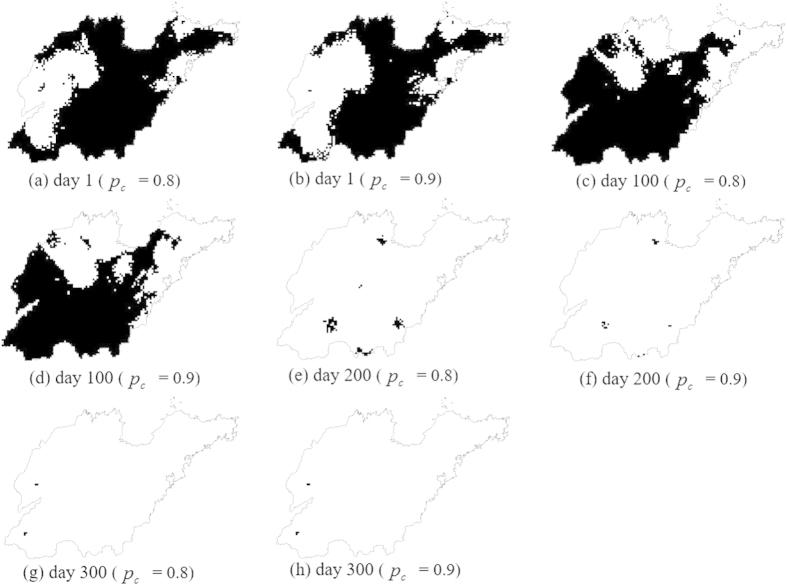
Contaminated sites determined by the conditions in Eq. (12) for (**a**) day 1 at *p*_*c*_ = 0.8; (**b**) day 1 at *p*_*c*_ = 0.9 (**c**) day 100 at *p*_*c*_ = 0.8; (**d**) day 100 at *p*_*c*_ = 0.9; (**e**) day 200 at *p*_*c*_ = 0.8; (**f**) day 200 at *p*_*c*_ = 0.9; (**g**) day 300 at *p*_*c*_ = 0.8 and (**h**) day 300 at *p*_*c*_ = 0.9. (Created by ArcMap, version 10.2, http://www.esri.com/).

**Figure 10:**
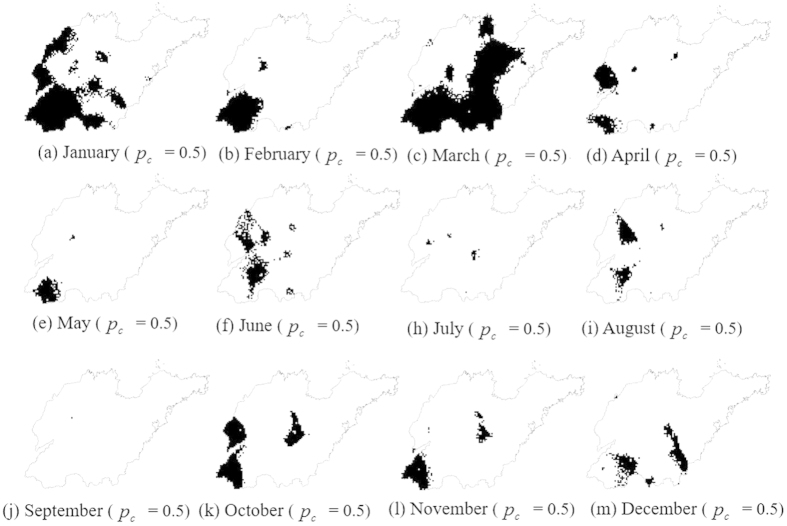
Contaminated sites determined by the conditions in Eq. (13) for each month (*p*_*c*_ = 0.5). (Created by ArcMap, version 10.2, http://www.esri.com/).

Figure 9 shows two types of maps for different days classified as contaminated based on the probability maps determined by


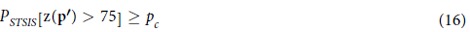


where the critical probabilities *p*_*c*_ = 0.9 and 0.8. Figure 10 shows the maps for every month classified as contaminated based on the exceedance probability maps determined by


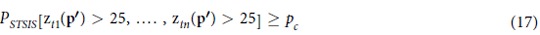


where the critical probability *p*_*c*_ = 0.5.

Tables 3 and 4 list the multi-location uncertainties for different days and different months (given *p*_*c*_) expressed by the corresponding *p*_*j*_ (i.e., the joint probabilities of the PM_2.5_ concentrations at *m* simulated locations of the contaminated sites all exceeding 75 μg · *m*^−3^ over different days). The associated variances 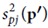 of Eq. (10) are also listed. The *p*_*j*_ value can be used to represent the reliability of the contaminated site classification. For example, the joint probability *p*_*j*_ is 0.76 based on 9234 simulated locations of the contaminated sites based on a given critical probability *p*_*c*_ = 0.8 for day 1, which means that for day 1, the probability of the PM_2.5_ concentration at all 9234 simulated locations exceeding the threshold (75 μg · *m*^−3^) is 76%. If the critical probability *p*_*c*_ = 0.9 is adopted, the joint probability is *p*_*j*_ = 0.9 in day 1, and the likelihood that the PM_2.5_ concentrations in the contaminated area will exceed 75 μg · *m*^−3^ is greater. However, the joint probabilities of all months are *p*_*j*_ = 0, indicating a zero likelihood that the PM_2.5_ concentrations at all of the simulated locations will exceed 25 μg · *m*^−3^ for each month.

### Goodness of uncertainty assessment: STSIS vs. SIS

To assess the improvements provided by using multi-temporal data, we first applied the purely spatial SIS technique to the original data recorded for each day of interest. In addition, to determine the effect of performing a composite spatiotemporal data analysis, we used the STSIS technique. The results of the SIS and STSIS techniques were assessed using the methods introduced in section 2.4. The CCDFs were obtained using the SIS and STSIS techniques and a cross validation of the monitoring points recorded during 2014.

**Figure 11:**
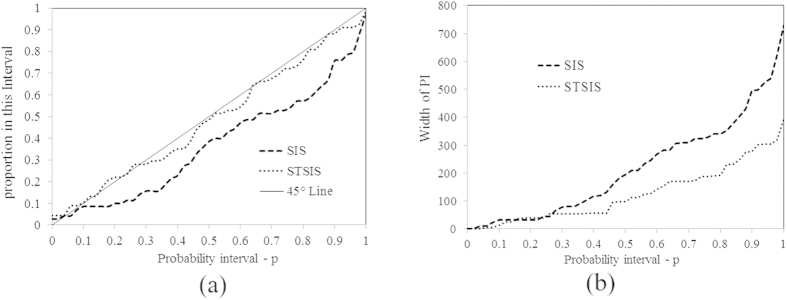
Plots of the (**a**) proportion of the actual PM_2.5_ concentrations falling within the PIs (accuracy plot); and the (**b**) PI widths vs. probability interval *p*. The STSIS and SIS algorithms were used to generate the CCDF models using cross validation.

As shown in Fig. 11(a), the distance between the estimated points on the plots and the 45° line was smaller for the STSIS technique than for the SIS techniques, and the RMSE values of Eq. (14) for the STSIS and SIS were 0.031 and 0.14, respectively. Hence, the STSIS probability analysis is more accurate than the SIS analysis. Figure 11(b) shows the PI widths, which in this case correspond to the differences between the maximum value and the (1-*p*)-quintile of the CCDF. All of the points of the STSIS analysis fall below the points of the SIS analysis, indicating that the PIs obtained by the STSIS are narrower than those of the SIS. Thus, the STSIS performs better than the SIS.

## Conclusions

In this work, the STSIS technique was used to perform uncertainty assessments for the PM_2.5_ concentrations in Shandong Province, China. The results suggest that the STSIS can represent composite spatiotemporal variations of PM_2.5_ concentrations using a non-separable semivariogram model as well as assimilate multi-temporal monitoring data.

A comparison of the results of the STSIS with that of the STOK showed that the map of PM_2.5_ concentrations generated by the STSIS exhibits more realistic variations and is closer to the experimental data than the map generated by the STOK. In addition, the PM_2.5_ maps for 2014 revealed marked spatial and temporal trends. In terms of the spatial trends, the western part of the study area was heavily polluted with PM_2.5_, whereas the eastern part of the study area presented relatively good air quality. In terms of the temporal trends, a significant seasonal trend was observed, with high concentrations observed in spring and winter and relatively low concentrations observed in summer and autumn.

The STSIS realizations can be used to determine various types of site classification uncertainties in terms of exceedance probabilities, including single-location/single-day uncertainties, single-location/multi-day uncertainties, multi-location/single-day uncertainties, and multi-location/multi-day uncertainties. A comparative analysis showed that by using multi-temporal data, the STSIS provided a better performance than the SIS because the corresponding probability intervals of the STSIS were consistently narrower than those of the SIS.

## Additional Information

**How to cite this article**: Yang, Y. *et al.* Uncertainty assessment of PM_2.5_ contamination mapping using spatiotemporal sequential indicator simulations and multi-temporal monitoring data. *Sci. Rep.* **6**, 24335; doi: 10.1038/srep24335 (2016).

## Figures and Tables

**Table 1:** Parameters of the non-separable spatiotemporal semivariogram model.

	*c*_0_	*c*	*v*	*w*	*ξ*	*α*	RMSE
*z*_*c*1_	0.0299	0.1188	0.6298	150000	148900	89980	0.0259
*z*_*c*2_	0.03384	0.1739	0.7331	150000	150000	90000	0.0131
*z*_*c*3_	0.03338	0.1663	0.1	150000	150000	135200	0.0159
*z*_*c*4_	0.02604	0.09751	0.1045	150000	150000	128400	0.0175
Lg(PM_2.5_)	0.04135	0.3132	97.26	3219000	530700	231400	0.0299

**Table 2:** PM_2.5_ concentration summary statistics of (a) the original data, (b) the STOK estimates, and (c) the three randomly selected STSIS realizations (out of 1000 realizations).

Method	Min	Max	Mean	SD	CV	Skewness	Kurtosis
STOK	0.84	538.16	79.77	49.01	0.61	1.722	4.649
STSIS_162	1	993.11	76.89	56.53	0.73	2.144	3.393
STSIS_369	1	992.77	75.86	60.39	0.80	2.143	3.387
STSIS_489	1	992.61	77.02	58.36	0.76	2.138	3.36
Original	1	1000	74.84	51.7	0.69	2.31	14.95

SD: standard deviation; CV: coefficient of variation.

**Table 3:** Uncertainty assessment of the sites where the PM_2.5_ concentrations are >75 μg · *m* ^−3^ based on the joint probabilities.

day	p_c_	m	p_j_	s_pj_
1	0.9	7724	0.9	0.03
	0.8	9234	0.76	0.04
100	0.9	5533	0.88	0.03
	0.8	6405	0.76	0.04
200	0.9	49	0.29	0.05
	0.8	162	0.09	0.03
300	0.9	5	1	0.00
	0.8	5	1	0.00

**Table 4:** Uncertainty assessment of the sites where the PM_2.5_ concentrations are >25 *mg*/*Kg* based on the joint probabilities.

Month	p_c_	m	p_j_	s_pj_
January	0.5	3976	0	0
February	0.5	1150	0	0
March	0.5	4236	0	0
April	0.5	1174	0	0
May	0.5	331	0	0
June	0.5	1048	0	0
July	0.5	38	0	0
August	0.5	315	0	0
September	0.5	1	0	0
October	0.5	1628	0	0
November	0.5	798	0	0
December	0.5	648	0	0
